# Elevated plasma levels of cardiac troponin-I predict left ventricular systolic dysfunction in patients with myotonic dystrophy type 1: A multicentre cohort follow-up study

**DOI:** 10.1371/journal.pone.0174166

**Published:** 2017-03-21

**Authors:** Mark J. Hamilton, Yvonne Robb, Sarah Cumming, Helen Gregory, Alexis Duncan, Monika Rahman, Anne McKeown, Catherine McWilliam, John Dean, Alison Wilcox, Maria E. Farrugia, Anneli Cooper, Josephine McGhie, Berit Adam, Richard Petty, The Scottish Myotonic Dystrophy Consortium, Cheryl Longman, Iain Findlay, Alan Japp, Darren G. Monckton, Martin A. Denvir

**Affiliations:** 1 West of Scotland Clinical Genetics Service, Queen Elizabeth University Hospital, Glasgow, United Kingdom; 2 Institute of Molecular, Cell and Systems Biology, College of Medical, Veterinary and Life Sciences, University of Glasgow, Glasgow, United Kingdom; 3 Clinical Genetics Service, Western General Hospital, Edinburgh, United Kingdom; 4 North of Scotland Clinical Genetics Service, Ashgrove House, Foresterhill Health Campus, Aberdeen, United Kingdom; 5 Human Genetics Unit, Ninewells Hospital, Dundee, United Kingdom; 6 Department of Neurology, Institute of Neurological Sciences, Queen Elizabeth University Hospital, Glasgow, United Kingdom; 7 Inherited Cardiac Conditions Service, Queen Elizabeth University Hospital, Glasgow, United Kingdom; 8 Centre for Cardiovascular Science, Queens Medical Research Institute, University of Edinburgh, Edinburgh, United Kingdom; University of Valencia, SPAIN

## Abstract

**Objective:**

High sensitivity plasma cardiac troponin-I (cTnI) is emerging as a strong predictor of cardiac events in a variety of settings. We have explored its utility in patients with myotonic dystrophy type 1 (DM1).

**Methods:**

117 patients with DM1 were recruited from routine outpatient clinics across three health boards. A single measurement of cTnI was made using the ARCHITECT *STAT* Troponin I assay. Demographic, ECG, echocardiographic and other clinical data were obtained from electronic medical records. Follow up was for a mean of 23 months.

**Results:**

Fifty five females and 62 males (mean age 47.7 years) were included. Complete data were available for ECG in 107, echocardiography in 53. Muscle Impairment Rating Scale score was recorded for all patients. A highly significant excess (*p* = 0.0007) of DM1 patients presented with cTnI levels greater than the 99^th^ centile of the range usually observed in the general population (9 patients; 7.6%). Three patients with elevated troponin were found to have left ventricular systolic dysfunction (LVSD), compared with four of those with normal range cTnI (33.3% versus 3.7%; *p* = 0.001). Sixty two patients had a cTnI level < 5ng/L, of whom only one had documented evidence of LVSD. Elevated cTnI was not predictive of severe conduction abnormalities on ECG, or presence of a cardiac device, nor did cTnI level correlate with muscle strength expressed by Muscle Impairment Rating Scale score.

**Conclusions:**

Plasma cTnI is highly elevated in some ambulatory patients with DM1 and shows promise as a tool to aid cardiac risk stratification, possibly by detecting myocardial involvement. Further studies with larger patient numbers are warranted to assess its utility in this setting.

## Introduction

Myotonic dystrophy type 1 (DM1) is a dominantly inherited, multisystem condition affecting approximately 1 in 8,000 of the population.[1] It is caused by expansion of a CTG trinucleotide repeat in the 3′-untranslated region of the *DMPK* gene. The trinucleotide repeat is genetically unstable in the germline, with offspring frequently inheriting a larger repeat expansion than the transmitting parent.[2] This gives rise to the phenomenon of genetic anticipation, with earlier onset and more severe symptoms often seen in successive generations of an affected family. Cardiac manifestations of DM1 include cardiomyopathy, conducting system disease and arrhythmias.[3, 4] Non-cardiac features include frontal balding, ptosis, skeletal muscle weakness, delayed muscle relaxation, diabetes and cognitive deficits.[5] Premature mortality is most commonly due to cardiac arrhythmias and respiratory failure.[6, 7] Abnormalities of the surface electrocardiogram are common, and include prolongation of the PR interval, axis-deviation and broadening of the QRS complex.[8] Arrhythmias may also occur including atrial fibrillation and flutter, ventricular tachycardia and varying degrees of atrioventricular block. In several case series, advanced cardiac involvement of the conducting system was linked to sudden death and cardiovascular events.[9–11] Overt cardiomyopathy is relatively rare, although cardiac MR studies suggest that more subtle changes in myocardial trabeculation and mass are more common than previously recognized.[8, 12, 13]

Cardiology follow up of patients with DM1 is often requested by clinical neurology or genetics teams before the appearance of clear cardiac involvement. The 12 lead ECG is commonly used as a screening tool, sometimes in association with 24 hour ambulatory monitoring and echocardiography[14] to identify those at higher risk of future cardiac events, or those who may benefit from treatment, such as patients with severe asymptomatic LVSD. Even in the absence of symptomatic dysrhythmia, insertion of a permanent cardiac pacemaker or implantable cardioverter defibrillator may be indicated in patients with high grade atrioventricular block or ventricular arrhythmias.[4] There is some evidence that the presence of left ventricular systolic impairment is associated with a worse prognosis.[10]

Plasma cardiac troponin has emerged as a useful clinical tool to identify patients at increased risk of cardiac events in a range of clinical settings.[15–18] We hypothesised that plasma cardiac troponin I (cTnI) levels may be elevated and act as a biomarker of cardiac involvement in DM1, and thus could prove a useful tool for risk stratification in this group.

## Methods

### Ethical considerations

The study, including consenting procedure, was reviewed by the West of Scotland Research Ethics Committee (reference 08/S0703/121). Multi-centre ethical approval was granted, and patients were recruited from three health authority regions in Scotland (NHS Greater Glasgow and Clyde, NHS Lothian, and NHS Grampian). All participants gave written, informed consent.

### Patient recruitment

Patients were identified during routine follow-up appointments at cardiology, clinical genetics and neurology outpatient clinics by their usual care team. Eligibility was defined as any patient with a genetically confirmed diagnosis of myotonic dystrophy type 1. Patients with a recent hospital admission with chest pain or a suspected cardiac event, as well as those currently experiencing angina or cardiac-type chest pains were excluded. Patients were not excluded on the basis of additional comorbidities such as diabetes or hypertension. No patients were known to have significant renal impairment. Demographic, clinical, electrocardiogram (ECG), echocardiogram (echo) data collected as part of routine clinical follow-up were obtained from electronic medical records. Muscle Impairment Rating Scale (MIRS) scores, routinely documented during annual review appointments, were also collected. The MIRS is an ordinal five-point rating scale of muscle impairment, validated specifically in DM1 (1 = no impairment; 2 = minimal signs; 3 = distal weakness; 4 = mild to moderate proximal weakness; 5 = severe proximal weakness). [19]

### Blood sampling and troponin assay

Whole blood was sent directly to the clinical biochemistry department for troponin analysis, or samples were centrifuged at 1000 x *g* for 15 minutes and stored at -40°C before being transported frozen for analysis. Cardiac TnI was measured using a high sensitivity assay (ARCHITECT *STAT* Troponin I assay; Abbott Laboratories). This assay has a limit of detection of 1.2 ng/L, with a coefficient of variation of 23% at the limit of detection (1.2 ng/L) and less than 10% at 6 ng/L. [20] The upper reference limit, determined by the manufacturer as the 99^th^ centile of samples from 4590 presumed healthy individuals, is 34 ng/L in men and 16 ng/L in women.[21] This is consistent with studies in other healthy populations.[22, 23]

### Genetic analysis

CTG repeat expansion sizes were estimated by small pool PCR as previously described.[24] Four reactions, each using 300 pg blood genomic DNA template, were performed for each patient. CTG repeat lengths were estimated by comparison against DNA fragments of known length in the molecular weight marker. The lower boundary of the expanded molecules in small pool PCRs using blood DNA was used to estimate the inherited, or “progenitor”, repeat length, [24] which is the major determinant of age at onset of symptoms.[25]

Echocardiography studies were performed in centres accredited by the British Society of Echocardiography, and conducted according to national protocols.[26]

### Statistics

Linear regression analyses were undertaken using SPSS for Macintosh software (Version 22.0. Armonk, NY; IBM Corp.). Two-tailed Fisher’s exact test was performed using GraphPad QuickCalcs website (La Jolla, California, USA. http://graphpad.com/quickcalcs/contingency1.cfm; accessed May 2016).

## Results

### Patient cohort

One hundred and seventeen patients with a diagnosis of DM1 were recruited and had cTnI level measured. These included 55 females and 62 males, with a mean age of 47.7 years (range of 21 to 72). Demographic and clinical data relating to diagnosis, presence of implanted cardiac device, associated comorbidity and MIRS score contemporaneous to cTnI sampling were available for the entire cohort. Complete ECG data were available for 107 patients, with partial information (*eg*. clinic letter description) for a further three. Echo data were available for 53 patients. Patients were followed up for a mean of 23 months (range 7 to 32 months) after cTnI testing.

Cardiac devices (including permanent pacemaker, internal cardioverter defibrillator or cardiac resynchronisation therapy) had been previously implanted in 21 patients. Echo had been carried out as part of normal clinical care within the previous 36 months and/or during the follow-up period in 53 patients. ECG data were available within 12 months for 107 individuals. Genetic data (estimated progenitor allele length and modal allele lengths) were available for 111 individuals.

### Outcomes

Seven patients died during the follow-up period. Three were sudden deaths, two of which were attributed to ischaemic heart disease (Table 1).

**Table 1:** Details of patient deaths during study period.

Study number (sex)	Age at death	cTnI (ng/L)	Comorbidities	Sudden death of suspected cardiac cause?	Details
DMGV1 (F)	67	18	Permanent pacemaker, concentric left ventricular hypertrophy	No	Aspiration event during evening meal.
DMGV105 (M)	47	2	Diabetes mellitus type 2	Yes	Unable to obtain detailed information. Recent admission with type II respiratory failure. Death certificate gives cause of death as hypoxic brain injury due to out-of-hospital cardiac arrest.
DMGV117 (F)	66	28	Dilated cardiomyopathy, mitral regurgitation, coronary artery disease, atrial fibrillation, dyslipidaemia	Yes	Witnessed sudden collapse. Ambulance crew confirmed VF arrest. ECG during attempted resuscitation in hospital consistent with extensive myocardial infarction.
DMGV129 (M)	71	5	Polycythaemia, right frontal lobe cerebral infarction, previous anteroseptal myocardial infarction.	Yes	Sudden death. Post-mortem examination concluded cause to be coronary artery disease.
DMGV141 (F)	58	9	Endometrial adenocarcinoma, poor mobility	No	Admitted to hospital with ankle fracture. Gradual deterioration over several days with type II respiratory failure, paroxysmal atrial fibrillation and confusion. Developed in-hospital cardiac arrest with complete heart block. Attempts at resuscitation were unsuccessful.
DMGV187 (M)	70	7	Cerebrovascular disease, permanent pacemaker, falls, poor cough.	No	Multiple admissions with deterioration in mobility and confusion, attributed to aspiration pneumonia. Insertion of a percutaneous gastrostomy was discussed, which the patient declined. Subsequently died at home with palliative care involvement.
DMGV227 (M)	68	8	Used non-invasive ventilation overnight	No	Aspiration pneumonia

### Age and muscle power at sampling

Previous authors have observed a positive correlation between age and increasing cTnI level in a population-based cohort.[27] We therefore used linear regression analysis to investigate the relationship between age and cTnI in our DM1 cohort, revealing a positive correlation overall (*p* = 0.037). This correlation was greater in female patients only (*p* = 0.022), but was not significant among male patients alone (*p* = 0.726).

Troponin level did not correlate with muscle power as measured by MIRS score in the population as a whole (*p* = 0.952), nor in male or female-specific sub-analyses (*p* = 0.801 and *p* = 0.783 respectively).

### Elevated troponin

Using sex-specific thresholds to define the upper limit of the expected cTnI range (34ng/L in males, 16ng/L in females), nine patients in our cohort (three males, six females) had elevated cTnI (Table 2). This confirms that substantial cTnI elevations occur significantly more frequently in DM1 patients compared with the manufacturertlycontrol population (*p* = 0.0007 in two-tailed Fisheril exact test).[21] Echo data were available for five patients with elevated cTnI, of whom three showed LV impairment (one mild, one moderate, one severe), and one mild LV hypertrophy. As shown in Table 2, the documented prevalence of LVSD was substantially higher in patients with elevated cTnI compared to those with normal cTnI, although rates of device implantation were similar. There was a trend toward increased mortality in the high cTnI group that did not reach statistical significance. Among the nine patients with cTnI > 99^th^ centile, four had a MIRS score of 4/5, four 3/5 and one 2/5.

**Table 2:** Prevalence of left ventricular dysfunction, device implantation and death during follow-up in DM1 patients with cTnI above population 99^th^ centile compared to those with troponin in the population normal range.

	cTnI > general population 99^th^ centile (n. 9)	cTnI in population normal range (n. 108)	*p*
Proven LV dysfunction	3 (33.3%)	4 (3.7%)	0.010
Pacemaker or ICD	2 (22.2%)	19 (17.6%)	0.663
Death during follow-up	2 (22.2%)	5 (4.6%)	0.091

### Low troponin

A recent prospective cohort study demonstrated a strong negative predictive value of troponin level < 5 ng/L in patients presenting with suspected acute coronary syndrome.[20] In our cohort, 62/117 patients had troponin < 5 ng/L (Table 3). Echo data were available for 26, of whom only one had LV dysfunction, which was considered borderline low with an ejection fraction of 49%. A further one patient had mild LV hypertrophy. The difference in prevalence of LVSD compared to all others in the cohort approached statistical significance (*p* = 0.0502). The average MIRS score of patients with cTnI < 5 ng/L was 3.41/5 (range 1 to 5), compared with 3.38/5 (range 2 to 5) among those with cTnI TnI nge. Three patients with troponin in the normal range but lence of LVSD compared to all oECG data were available for two of these, both males, who had normal PR and QRS values, and QTc of 410 ms and 438 ms respectively.

**Table 3:** Prevalence of left ventricular dysfunction, device implantation and death during follow-up in DM1 patients with cTnI < 5 ng/L compared to those with cTnI ≥5ng/L.

	cTnI < 5 ng/L (n. 62)	cTnI ≥ 5 ng/L (n. 55)	*p*
Proven LV dysfunction	1 (1.6%)	6 (10.9%)	0.050
Pacemaker or ICD	12 (19.4%)	9 (16.4%)	0.810
Death during follow-up	1 (1.6%)	6 (10.9%)	0.050

Only one patient with cTnI < 5ng/L died during the follow-up period. The limited data available suggest the cause of death was an out-of-hospital cardiac arrest, although the patient had a recent hospital admission with type II respiratory failure.

### Conducting system disease and Groh criteria

Among DM1 patients, the presence of PR interval > 240 ms, QRS > 120 ms, atrial tachyarrhythmias and/or any rhythm other than sinus (Groh criteria) have previously been shown to predict sudden death.[4, 9]

Thirty one patients in our cohort met Groh ECG criteria, including five with PR > 240ms and 18 with QRS > 120 ms. Ten patients were not in sinus rhythm; three in atrial fibrillation, three in atrial flutter and four with a pacemaker-dependent rhythm. Neither elevated cTnI nor cTnI < 5 ng/L predicted the presence of high risk ECG changes (Table 4). There was a trend towards increased mortality among patient with any high risk ECG features that did not reach statistical significance, and non-sinus rhythm ECG was present in all three patients meeting Groh criteria who died during follow-up (*p* = 0.013).

**Table 4:** Clinical features, cTnI levels and outcome in patients with high risk ECG features compared to those without.

	High risk ECG features present (n. 31)	No high risk ECG features recorded (n. 86)	*p*
Proven LV dysfunction	2 (6.5%)	5 (5.8%)	1.000
Pacemaker or ICD	13 (41.9%)	8 (9.3%)	< 0.001
Death during follow-up	3 (9.7%)	4 (4.7%)	0.380
cTnI > population 99^th^ centile	3 (9.7%)	6 (7.0%)	0.698
cTnI < 5 ng/L	14 (45.2%)	48 (55.8%)	0.402

ECG data were available for four of the five patients with LVSD who did not meet Groh criteria. PR duration was normal in two, borderline in two (198 ms and 204 ms respectively), and QTc ≤ 438 ms in all. Four of these five patients had cTnI ≥ 5 ng/L, including one above the population 99^th^ centile.

Linear regression analysis showed no significant correlation between cTnI measurement and either PR interval or QRS duration for the cohort as a whole. Likewise correlations between PR interval and QRS duration themselves were not significant. Sub-analysis in male patients only, however, revealed a significant positive correlation between PR interval and cTnI level (*p* = 0.025) and a strong correlation between QRS duration and PR interval (*p* = 0.002). These were non-significant in female patients (*p* = 0.682 and *p* = 0.992 respectively).

### Genetic data

The cohort was highly genetically heterogeneous, both in terms of modal CTG repeat length (range 46 to 1264 repeats, mean 442 repeats) and estimated progenitor allele length (range 43 to 730 repeats, mean 222 repeats).

No correlation was observed between cTnI level and progenitor allele length nor modal allele length in univariate analysis of the cohort as a whole. This was also true in multivariate analysis integrating both age at sampling and allele size. In sex-specific sub-analysis, only the correlation between cTnI and modal allele length in female patients approached statistical significance (*p* = 0.079).

## Discussion

In clinical practice, the role of plasma cardiac troponin measurement as a screen for myocardial necrosis in the context of acute chest pain is well established. However, high-sensitivity cardiac troponin assays have also shown promise as prognostic biomarkers of cardiovascular risk in a variety of ambulatory outpatient settings, including among those with known vascular disease,[28, 29] the elderly [30, 31] and in general population-based studies.[27, 32–35] The cause of chronic, low-level troponin elevation in these populations is uncertain, although speculated mechanisms include subclinical ischaemia, microvascular dysfunction or apoptosis of myocytes.[36]

In this context, we present exploratory data relating to cTnI from 117 patients with DM1, recruited from those attending routine review appointments. Remarkably, we found that 9 (7.6%) have a cTnI above the general population 99^th^ centile. This strongly suggests that cTnI levels act as a marker of a component of the DM1 disease process, although the precise clinical significance of elevated cTnI and the pathological processes that it represents in this group are unclear.

Elevated levels of the T subunit of cardiac troponin (cTnT) have previously been reported in patients with a range of myopathic conditions. The origin of elevated cTnT in these cases is not fully resolved, although broadly they were not accompanied by an increase in cTnI except in some patients with concomitant cardiomyopathy [37–42]. There is some direct evidence that cTnT may be produced by regenerating skeletal muscle.[43] Very recently, Valaperta *et al* explored a range of cardiac biomarkers in a mixed cohort of 59 patients with DM1 or myotonic dystrophy type 2, comparing to both unaffected controls and controls with comparable cardiac morbidity. They found significantly higher levels of both serum cTnT and cTnI in those with myotonic dystrophy compared with controls, and a weakly significant association of both measures with disease duration. Skeletal muscle biopsy revealed expression of cTnT at the mRNA level in both healthy and DM1 muscle, and expression at protein level was detected by M7 detector antibody, though negative with 5D8 capture antibody. No measures of cTnI in this cohort were above the given cut-off value (26 ng/L; the non-sex adjusted population 99^th^ centile), and there were only two patients with manifest left ventricular impairment, limiting analysis with respect to heart muscle disease [44].

To our knowledge, there is no evidence that cTnI is expressed in healthy or diseased skeletal muscle.[45, 46] In the cohort we present, the association observed between cTnI level and presence of LV impairment, significant correlation with PR interval in male-specific subanalysis and the absence of correlations with muscle impairment as measured by MIRS score all strongly support a cardiac origin of the cTnI detected.

The 12-lead ECG currently serves as the cornerstone of cardiac assessment in DM1, with Groh criteria (PR > 240 ms, QRS > 120 ms, rhythm other than sinus, second-degree or third-degree AV block) shown to be predictive of sudden death and cardiac morbidity.[4, 9] Our data suggest that an elevated cTnI does not reliably distinguish patients with severe conduction abnormalities; 31 patients had high-risk ECG changes, among whom only three had elevated cTnI. Conversely however, we identified at least four patients with significant LVSD who would not be identified by Groh criteria on surface ECG alone, of whom three had cTnI ≥ 5 ng/L. Our findings therefore suggest that cTnI level represents a component of the cardiac phenotype distinct from progressive conducting system disease, apparently mapping more closely with myocardial involvement. This supports a potential role for combined ECG and biomarker measurement in cardiac screening for patients with DM1.

Patients with DM1 also have a high prevalence of risk factors for premature vascular disease, including insulin resistance,[47] obesity, and sedentary lifestyle.[48, 49] Despite this, risk from cardiovascular disease in DM1 is a relatively neglected area of investigation, with the vast majority of literature focused on DM1-specific risk of arrhythmia. We observed two deaths attributed to ischaemic heart disease in our cohort, one of whom had been found to have an elevated cTnI. This highlights coronary heart disease as a possible additional important cause of death in DM1 patients, and suggests value in further investigation of high-sensitivity troponin assay not only as a marker of DM1-specific heart disease, but also of more general cardiovascular risk in this group of patients.

We did not observe any correlation between cTnI level and CTG repeat length, expressed either as modal allele length or estimated progenitor allele length. This remained true in multivariate analyses to account for the patient’s age at troponin sampling. This is despite the small-pool PCR techniques used to measure the CTG repeat in this study having been shown to be superior in detecting genotype-phenotype correlations compared with traditional methods.[25] The absence of direct correlation between cTnI and genotype would however support cTnI as a marker of a multifactorial process, rather than a primary effect of the CTG repeat expansion on cardiac myocyte metabolism. Of note, elevated cTnI was not seen in any patients with an estimated progenitor allele length greater than 363 repeats. Larger CTG expansions are associated with shorter life expectancy in DM1,[50] and those with the severe, congenital onset form are much more likely to die from respiratory complications than cardiac arrhythmia, cardiomyopathy or sudden unexplained death.[51] The absence of high cTnI levels in those with very large repeat expansions therefore may simply reflect a lower burden of progressive cardiac disease due to reduced long-term survival.

Strengths of this study include sampling of cTnI from a large cohort of individuals with DM1, recruited from routine outpatient clinics across three different health regions, including annual review appointments offered to all individuals with DM1 known to their regional Clinical Genetics service. There is therefore little sampling bias in terms of severity of cardiac phenotype. The study is however significantly limited by a lack of a formal prospective clinical and cardiac review linked to the blood sampling, with all ECG and echocardiogram data obtained from electronic records. Patients therefore only underwent investigations that were requested as part of routine clinical care, and in many cases these were not closely contemporaneous to blood sampling. As a result, clinical data were incomplete, with echocardiogram results in particular only available for 44%. The correlations we describe with LVSD therefore assume absence of heart muscle involvement in patients for whom echo data was not available. Furthermore, analysis of correlations with PR interval inevitably excludes the 10 patients described with atrial fibrillation, atrial flutter or pacemaker-dependent ECGs. Absence of ECG data for seven patients, including one with elevated cTnI and one who died during follow-up (DMGV227), means that some patients with potentially severe phenotypes were excluded from these analyses. Finally, although finding nine individuals with cTnI above the population 99^th^ centile is itself remarkable, this is a relatively small number in absolute terms for further analysis. We therefore present these results as exploratory data, suggesting value in further systematic analysis of cTnI and other cardiac biomarkers in DM1.

## Conclusions

In ambulatory patients with DM1, plasma cTnI level appears to map with the presence of LVSD but does not correlate with advanced conducting system disease on surface ECG. These findings suggest that high sensitivity cTnI holds promise as a tool to aid risk stratification of patients with myotonic dystrophy, possibly adding additional value to surface ECG in identifying those at increased risk of cardiovascular disease or those with manifest cardiac muscle involvement. Further studies of cardiac biomarkers in DM1 involving larger patient numbers and longer-term formal follow-up are therefore warranted.
