# Correction: Elevated plasma levels of cardiac troponin-I predict left ventricular systolic dysfunction in patients with myotonic dystrophy type 1: A multicentre cohort follow-up study

**DOI:** 10.1371/journal.pone.0175615

**Published:** 2017-04-06

**Authors:** Mark J. Hamilton, Yvonne Robb, Sarah Cumming, Helen Gregory, Alexis Duncan, Monika Rahman, Anne McKeown, Catherine McWilliam, John Dean, Alison Wilcox, Maria E. Farrugia, Anneli Cooper, Josephine McGhie, Berit Adam, Richard Petty, The Scottish Myotonic Dystrophy Consortium, Cheryl Longman, Iain Findlay, Alan Japp, Darren G. Monckton, Martin A. Denvir

There are typographical errors in the Results section of the published article.

The following sentence under is incorrect: “This confirms that substantial cTnI elevations occur significantly more frequently in DM1 patients compared with the manufacturertlycontrol population (p = 0.0007 in two-tailed Fisheril exact test).” The correct sentence is: “This confirms that substantial cTnI elevations occur significantly more frequently in DM1 patients compared with the manufacturer’s control population (p = 0.0007 in two-tailed Fisher’s exact test).”

The following sentence is incorrect: “The average MIRS score of patients with cTnI < 5 ng/L was 3.41/5 (range 1 to 5), compared with 3.38/5 (range 2 to 5) among those with cTnI TnI nge.” The correct sentence is: “The average MIRS score of patients with cTnI < 5 ng/L was 3.41/5 (range 1 to 5) compared with 3.38/5 (range 2 to 5) among those with cTnI ≥ 5ng/L.”

The following sentence is incorrect: “Three patients with troponin in the normal range but lence of LVSD compared to all oECG data were available for two of these, both males, who had normal PR and QRS values, and QTc of 410 ms and 438 ms respectively.” The correct sentence is: “Three patients with troponin in the normal range but ≥ 5ng/L had evidence of LVSD. ECG data were available for two of these, both males, who had normal PR and QRS values, and QTc of 410 ms and 438 ms respectively.”
